# Molecular Characteristics of Genes and the Immune Microenvironment of a Rare Chest Malignant Tumor (Pulmonary Clear Cell Sarcoma): A Case Report

**DOI:** 10.3389/fonc.2021.664883

**Published:** 2021-03-22

**Authors:** Xiaoling Xu, Ding Wang, Wei Wu, Hongyang Lu

**Affiliations:** ^1^ Department of Thoracic Oncology, Cancer Hospital of the University of Chinese Academy of Sciences (Zhejiang Cancer Hospital), Hangzhou, China; ^2^ Institute of Cancer and Basic Medicine (IBMC), Chinese Academy of Sciences, Hangzhou, China; ^3^ Department of Thoracic Surgery, Cancer Hospital of the University of Chinese Academy of Sciences (Zhejiang Cancer Hospital), Hangzhou, China; ^4^ Department of Pathology, Cancer Hospital of the University of Chinese Academy of Sciences (Zhejiang Cancer Hospital), Hangzhou, China; ^5^ Zhejiang Key Laboratory of Diagnosis & Treatment Technology on Thoracic Oncology (Lung and Esophagus), Cancer Hospital of the University of Chinese Academy of Sciences (Zhejiang Cancer Hospital), Hangzhou, China

**Keywords:** clear cell sarcoma, next-generation sequencing, lung, EWS-ATF1, PD-L1, TIGIT

## Abstract

Pulmonary clear cell sarcoma is a rare malignant tumor that has rarely been reported and is challenging to diagnose, especially when differentiating from malignant melanoma. Currently, EWSR1-ATF1 is the key marker for distinguishing clear cell sarcoma from melanoma, but IHC has diagnostic limitations. We report a patient diagnosed with pulmonary clear cell sarcoma, in which an NGS was used to help with the pathological diagnosis. The exposure to the immune microenvironment in pulmonary clear cell sarcoma suggests that TIGIT-related drugs may be a new and effective treatment for this rare disease. Immune microenvironment-related markers, including PD-L1, CD8, TIM3, LAG3, and CD163, were negatively expressed in pulmonary clear cell sarcoma.

## Introduction

Enzinger first described a rare soft tissue tumor of clear cell sarcoma (CCS) in 1965 that arose from the abnormal differentiation of pigment cells (1), previously known as soft tissue malignant melanoma. CCS cases account for approximately 1% of rare tumors originating from stromal cells (2). CCS is most common in young men and women between the ages of 20 and 40 years. CCS usually arises on the distal extremities, especially on the tendons and aponeurosis of the foot and ankle and on the arms, hands, and trunk, as reported by Goh et al. (3). At present, EWSR1-ATF1 is used as a key marker to distinguish melanoma from CCS (4). This disease has a slow course, and the average age of CCS diagnosis is 39 years. According to Gonzaga et al., at diagnosis, CCS is usually advanced and locally aggressive, with a high rate of recurrence and metastasis (up to 50%). The most common site of distant metastases is the lung; the overall 5- and 10-year survival rates are approximately 50 and 38%, respectively, with no significant differences in survival rates between males and females (5). Although surgical treatment may be beneficial for the patient, new molecular targeted therapy should be implemented to improve the oncologic outcome in early- and late-stage disease (2, 6). Hence, we report a patient with pulmonary CCS, in which NGS was used to help with the diagnosis and explore molecular characteristics of genes and the immune microenvironment of pulmonary CCS in this patient.

## Case Description

**Figure 1:**
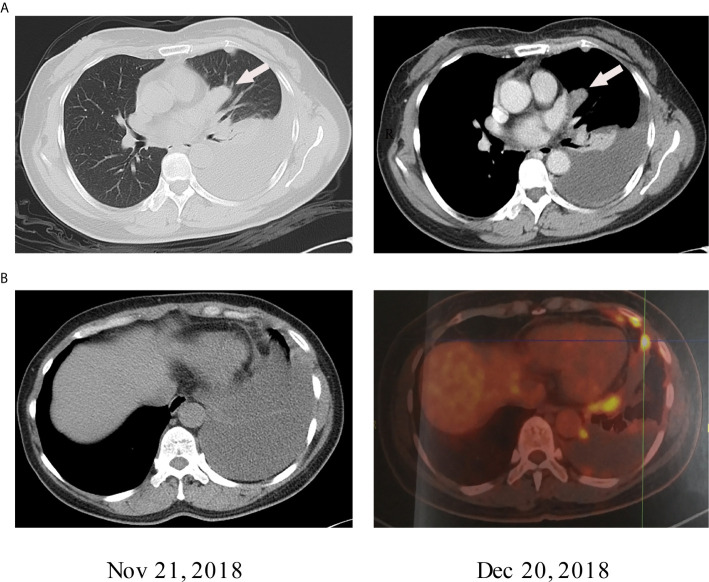
**(A)** The lung windows show 2.3 cm × 2 cm nodules with uniform density next to the heart margin of the left lung segment. Contrast-enhanced CT of mediastinal windows shows that the nodules were uniformly strengthened. **(B)** The PET-CT results of the patient on December 20, 2018, showed a decrease in pleural effusion compared to the PET-CT results on November 21, 2018.

**Figure 2:**
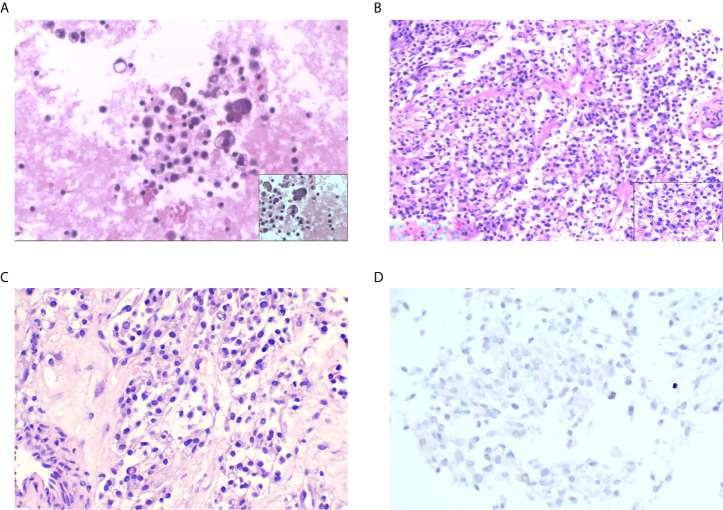
**(A)** Scattered lymphocytes, tissue cells, mesothelial cells, and individual atypical cells (H&E staining: original magnification ×100). **(B)** Epithelioid tumor cells distributed in nests and sheets around fibers and blood vessels; some of the cells have transparent cytoplasm with nuclear division (H&E staining: original magnification ×100). **(C)** PD-L1 control (H&E staining: original magnification ×100). **(D)** PD-L1-negative staining.

**Figure 3:**
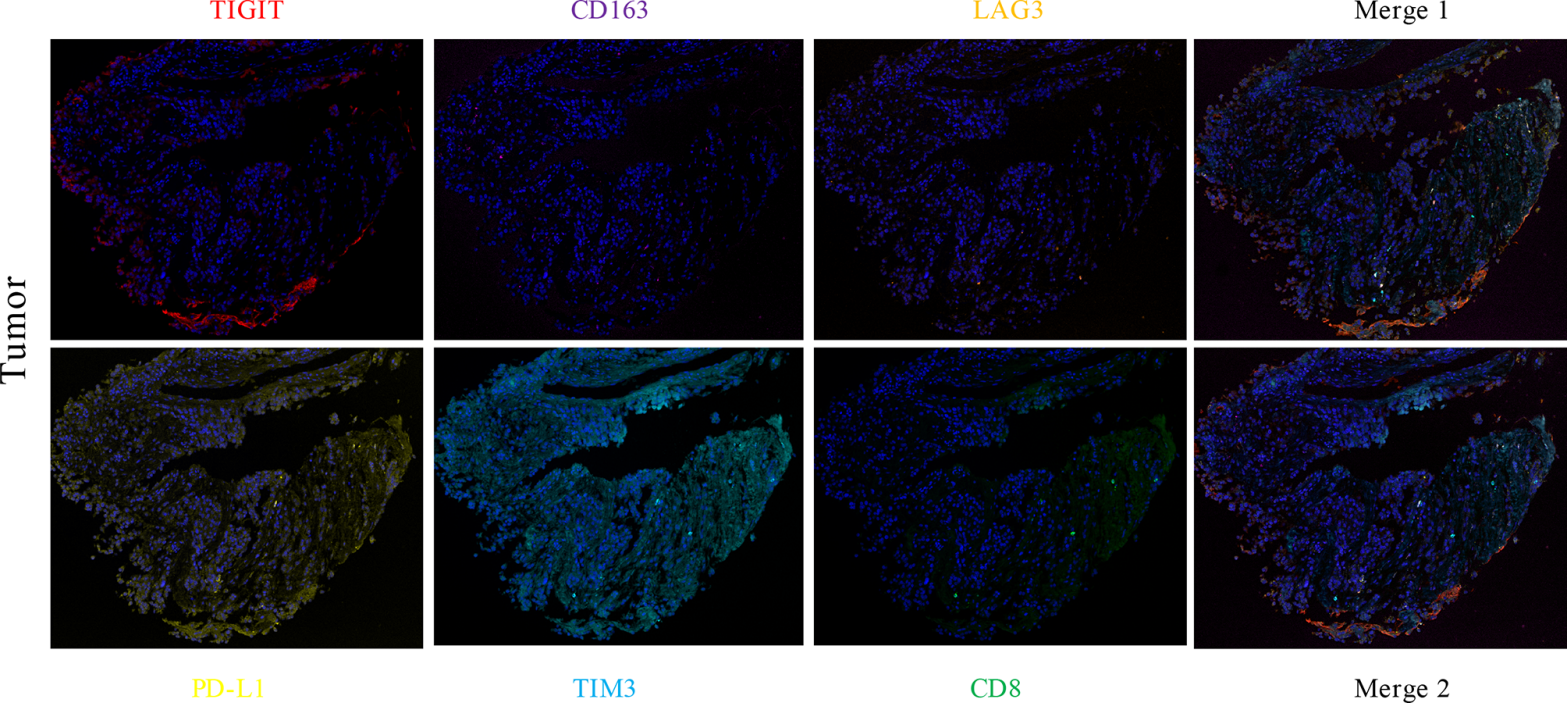
The expression levels of CD8 (green), TIM3 (light blue), PD-L1 (yellow), LAG3 (orange), CD163 (purple), and TIGIT (red) in lung CCS were detected by multiple immunofluorescence assays. The number of positive cells was estimated as follows: TIGIT ≥1%, other indexes **<**1%. CD8 and TIM3 colocalized, and there was no colocalization with other indexes.

A 51-year-old Chinese woman visited the hospital because of chest tightness for 10 days. Her chest CT showed a 2.3 cm × 2 cm nodule in her left lung. Multiple plaques were seen on the left pleura. Pleural effusion, compression atelectasis, and swollen hilar lymphadenopathy in the left lung can be seen on contrast-enhanced CT (**Figure 1A**). From November 19, 2018, after admission to the hospital, obvious tumor cells could not be found in the repeated pleural effusion tests (**Figure 2A**). Additionally, bronchoscopy, neck lymph nodes, and brain CT showed no apparent abnormalities. A biopsy of the left pleural mass was performed on November 20, 2018. The histopathologic findings revealed an epithelioid tumor with fibrous vascular nests and strands surrounding it. Some cells were hyaline, and mitotic figures were seen. Positive staining for Ki-67 (approximately 40%), Sy, Melan-A, Hmb45, and S-100 was detected by immunohistochemistry using monoclonal antibodies (**Figure 2B**). The patient had no previous skin lesions and had no history of previous excision of the skin or other lesions. We used bevacizumab 200 mg for left intrathoracic treatment on December 7, 2018, for patients with self-reported chest tightness improved with this treatment. Because this primary pathological type of lung disease is rare, we sent a pleural biopsy sample for the next-generation sequencing of tissue samples to FoundationOne CDX on December 14, 2018; NGS revealed an EWSR1-ATF1 fusion, CDKN2A/b loss, and MTAP loss (**Table 1**). We also confirmed that PD-L1 as a target by immunohistochemistry using an anti-PD-L1 IHC 22C3 pharmDx (Dako) antibody (**Figures 2C, D**). Because the PD-L1 target did not perform as expected, we performed immunofluorescence analysis (**Figure 3**) with the OPAL™ Multiplex IHC (Akoya) Kit. Immune microenvironment-related markers, including CD8, TIM3, PD-L1, LAG3, CD163, and TIGIT, were examined to find a new therapeutic target. Only TIGIT was observed in positive cells ≥1%, and there were <1% positive cells for the other markers. CD8 and TIM3 showed colocalization, but no colocalization was identified between the other markers. TIGIT is one of the most promising and potential targets in the new generation of immunotherapy drugs, and several anti-TIGIT monoclonal antibodies have been studied. The outcome of the PET-CT scan on December 20, 2018, showed that the patient’s pleural effusion improved after bevacizumab treatment (**Figure 1B**). However, the patient refused further treatment after the diagnosis of pulmonary CCS. Follow-up until her death on June 27, 2019.

**Table 1:** NGS panel findings.

Genomic findings	Lung tissue results	Biomarker findings	Lung tissue results
EWSR1	EWSR1-ATF1 fusion	Tumor mutational burden	TMB-low (3 mutations/Mb)
CDKN2A/B	Loss	Microsatellite status	MS-stable
MTAP	Loss		

## Discussion

CCS is a rare stromal soft tissue tumor similar to melanoma and soft tissue sarcoma but has a different genetic history. The clinical features of CCS lack specificity, which often manifests as slow painless local growth of the mass, and patients can experience local pain, itching, movement restrictions, easy postoperative recurrence, or metastasis (1, 7, 8). CT and MRI have limited diagnostic value for CCS, and CCS is confirmed by biopsy pathology and IHC staining; its variants often need molecular testing. The classic histological feature of soft tissue CCS is a small cluster of polygonal cells and spindle cells, characterized by hyaline to slightly basophilic cytoplasm and vesicular nuclei with prominent nucleoli separated by fine fibers (1, 9). Both CCS and malignant melanoma originate from melanocyte differentiation, so they are positive for common melanocyte markers such as S-100, HMB-45, MelanA, and NKI/C3 (2). We cannot easily distinguish them by immunohistochemistry. Hence, the use of fluorescence *in situ* hybridization or reverse transcription-polymerase chain reaction (RT-PCR) is essential for diagnosing CCS and distinguishing CCS from primary and metastatic melanoma (2). The cytogenetic feature of CCS is t (12; 22) (q13; q12), resulting in a chimeric EWSR1/ATF1 gene in which the EWS 3’ terminus at 22q is replaced by the ATF1 3’ terminus at 12q. Therefore, EWSR1/ATF1 can be used as a marker to distinguish CCS from melanoma (4). In this case, NGS was a feasible method to help pathologically diagnose CCS as the diagnosis was difficult to determine after biopsy and immunohistochemistry. The EWSR1-ATF1 fusion also supported the diagnosis of pulmonary CCS.

Yasmin Aghajan reported a case in which a novel EWSR1-ATF1 gene fusion was revealed using next-generation sequencing analysis, but FLI-1 immunohistochemical results were negative, suggesting that NGS has the advantage of showing the CCS EWSR1-ATF1 fusion (10). NGS was finally used to help diagnose this rare disease. Compared with traditional biopsy pathology and immunohistochemistry, NGS has unique advantages; NGS not only improves the diagnostic accuracy but also provides more possibilities for follow-up treatment strategies (11). Whereas the workload of NGS is still large, and the cost is still high. And the technical defects of NGS can also affect the final test result. In this study, the patient had a previously unknown CCS mutation, CDKN2AB, which was identified with NGS, and the absence of CDKN2AB may lead to hyperimmune progression (12). Moreover, we examined the PD-L1 expression and the immune microenvironment of this patient, which is the first time these were studied in CCS in the chest. However, this study also has obvious limitations. After the patient was diagnosed, we strongly recommended chemotherapy or immunotherapy, but the patient refused for economic reasons; therefore, we did not observe the patient’s efficacy. However, this study suggests that bevacizumab appears to have some effect in treating pleural effusion within the cavity with CCS.

Currently, the most effective treatment for most patients with CCS remains surgical resection, with only a small fraction of patients benefiting from conventional cytotoxic chemotherapy (6). Some of the latest potential therapeutic targets of CCS, such as MET, PDGFRA/B, and HDAC, have been identified. Due to the identification of these targets, some small molecules and monoclonal antibodies, such as sunitinib, sorafenib, and crizotinib, are in clinical trials. These treatments offer new hope for improving the prognosis of patients with this rare invasive disease (2, 13).

The immunophenotype of CCS is similar to that of melanoma, so CCS may have similar immunotherapy targets at similar immune checkpoints. PD-1- and PD-L1-associated antibodies have a wide range of antitumor effects and have been shown to benefit melanoma patients (14). The PD-L1 antibody as a potential immunotherapy for CCS was not favorable in our patient, suggesting that CCS may lack relevant immune checkpoints. In the meantime, we further explored the tumor microenvironment of the pulmonary CCS to search for more effective therapeutic targets with immunofluorescence. The results suggest that drugs that target TIGIT could become a new treatment for CCS. In 2009, TIGIT was first discovered by Xin Yu (15). TIGIT is considered a desirable target for cancer treatment because it can hinder the cancer immunity cycle’s multiple steps. Pre-clinical studies indicated that TIGIT blockade might protect against multiple solid and hematological cancers was confirmed in Pre-clinical studies (16). Several clinical trials (Phase 1, 2) of human anti-TIGIT mAbs are tested to treat advanced solid cancers, and its combination with PD-1 blockade enhances the antitumor effect (17). In the meantime, with the continuous advancement of clinical trials and our increasing understanding of the mechanism of TIGIT-mediated immune response regulation, more effective treatment strategies for cancer patients will emerge.

## Conclusion

In summary, pulmonary CCS is a rare soft tissue malignant tumor that occurs in young adults and has rarely been reported. In our study, a new NGS technique was used to help pathologically diagnose PCCS, thereby improving the diagnostic strategy, especially the differential diagnosis between CCS and malignant melanoma. Exploring the molecular characteristics of genes and the immune microenvironment of pulmonary CCS will be an essential for the clinical treatment of this rare disease

## Data Availability Statement

The original contributions presented in the study are included in the article/supplementary material. Further inquiries can be directed to the corresponding author.

## Ethics Statement

The studies involving human participants were reviewed and approved by the Ethics Committee of the Zhejiang Cancer Hospital. Written informed consent for participation was not required for this study in accordance with the national legislation and the institutional requirements.

## Author Contributions

XX and HL designed the study. DW and WW contributed to the data curation. HL supervised the study. XX and DW wrote the original draft of the manuscript. HL wrote, reviewed, and edited the manuscript. All authors contributed to the article and approved the submitted version.

## Acknowledgments

This study was supported by the Cancer Hospital of the University of Chinese Academy of Sciences (Zhejiang Cancer Hospital).

## Conflict of Interest

The authors declare that the research was conducted in the absence of any commercial or financial relationships that could be construed as a potential conflict of interest.

## References

[1] Enzinger FM. Clear-cell sarcoma of tendons and aponeuroses. An analysis of 21 cases. Cancer (1965) 18:1163–74. 10.1002/1097-0142(196509)18:9<1163::AID-CNCR2820180916>3.0.CO;2-0 14332545

[2] Cornillie J van Cann T Wozniak A Hompes D Schöffski P. Biology and management of clear cell sarcoma: state of the art and future perspectives. Expert Rev Anticancer Ther (2016) 16:839–45. 10.1080/14737140.2016.1197122 27253849

[3] Goh GH Teh M Vanecek T Moran C Petersson F. Primary pulmonary clear cell sarcoma-the first two reported cases. Virchows Arch (2016) 469:111–7. 10.1007/s00428-016-1943-8 27112339

[4] Hantschke M Mentzel T Rütten A Palmedo G Calonje E Lazar AJ. Cutaneous clear cell sarcoma: a clinicopathologic, immunohistochemical, and molecular analysis of 12 cases emphasizing its distinction from dermal melanoma. Am J Surg Pathol (2010) 34:216–22. 10.1097/PAS.0b013e3181c7d8b2 PMC5798433 20087159

[5] Gonzaga MI Grant L Curtin C Gootee J Silberstein P Voth E. The epidemiology and survivorship of clear cell sarcoma: a National Cancer Database (NCDB) review. J Cancer Res Clin Oncol (2018) 144:1711–6. 10.1007/s00432-018-2693-6 PMC11813523 29961184

[6] Bianchi G Charoenlap C Cocchi S Rani N Campagnoni S Righi A. Clear cell sarcoma of soft tissue: A retrospective review and analysis of 31 cases treated at Istituto Ortopedico Rizzoli. Eur J Surg Oncol (2014) 40:505–10. 10.1016/j.ejso.2014.01.016 24560887

[7] Lucas DR Nascimento AG Sim FH. Clear cell sarcoma of soft tissues. Mayo Clinic experience with 35 cases. Am J Surg Pathol (1992) 16:1197–204. 10.1097/00000478-199212000-00006 1463095

[8] Chung EB Enzinger FM. Malignant melanoma of soft parts. A reassessment of clear cell sarcoma. Am J Surg Pathol (1983) 7:405–13. 10.1097/00000478-198307000-00003 6614306

[9] Hisaoka M Ishida T Kuo T-T Matsuyama A Imamura T Nishida K. Clear cell sarcoma of soft tissue: a clinicopathologic, immunohistochemical, and molecular analysis of 33 cases. Am J Surg Pathol (2008) 32:452–60. 10.1097/PAS.0b013e31814b18fb 18300804

[10] Aghajan Y Malicki DM Levy ML Crawford JR. Atypical central neurocytoma with novel EWSR1-ATF1 fusion and MUTYH mutation detected by next-generation sequencing. BMJ Case Rep (2019) 12:1. 10.1136/bcr-2018-226455 PMC6340561 30642852

[11] Fernandez-Marmiesse A Gouveia S Couce ML. NGS Technologies as a Turning Point in Rare Disease Resea rch, Diagnosis and Treatment. Curr Med Chem (2018) 25:404–32. 10.2174/0929867324666170718101946 PMC5815091 28721829

[12] Giusti R Mazzotta M Filetti M Marinelli D Di Napoli A Scarpino S. CDKN2A/B gene loss and MDM2 alteration as a potential molecular signature for hyperprogressive disease in advanced NSCLC: A next-generation-sequencing approach. JCO (2019) 37:e20628–8. 10.1200/JCO.2019.37.15_suppl.e20628

[13] Mir O Boudou-Rouquette P Larousserie F Babinet A Dumaine V Anract P. Objective response to sorafenib in advanced clear-cell sarcoma. Ann Oncol (2012) 23:807–9. 10.1093/annonc/mds005 22274882

[14] Mahoney KM Freeman GJ McDermott DF. The Next Immune-Checkpoint Inhibitors: PD-1/PD-L1 Blockade in Melanoma. Clin Ther (2015) 37:764–82. 10.1016/j.clinthera.2015.02.018 PMC4497957 25823918

[15] Yu X Harden K Gonzalez LC Francesco M Chiang E Irving B. The surface protein TIGIT suppresses T cell activation by promoting the generation of mature immunoregulatory dendritic cells. Nat Immunol (2009) 10:48–57. 10.1038/ni.1674 19011627

[16] Harjunpää H Guillerey C. TIGIT as an emerging immune checkpoint. Clin Exp Immunol (2020) 200:108–19. 10.1111/cei.13407 PMC7160651 31828774

[17] Chauvin J-M Zarour HM. TIGIT in cancer immunotherapy. J Immunother Cancer (2020) 8:4. 10.1136/jitc-2020-000957 PMC7477968 32900861

